# A trade-off between oxidative stress resistance and DNA repair plays a role in the evolution of elevated mutation rates in bacteria

**DOI:** 10.1098/rspb.2013.0007

**Published:** 2013-04-22

**Authors:** Clara Torres-Barceló, Gabriel Cabot, Antonio Oliver, Angus Buckling, R. Craig MacLean

**Affiliations:** 1 Department of Zoology, University of Oxford, Oxford OX1 3PS, UK; 2 Servicio de Microbiología and Unidad de Investigación, Hospital Son Espases, Instituto Universitario de Investigación en Ciencias de la Salud (IUNICS), Palma de Mallorca, Spain; 3 Department of Bioscience, University of Exeter, Penryn TR10 9EZ, UK

**Keywords:** mutator, *Pseudomonas aeruginosa*, oxidative stress, evolution, trade-off

## Abstract

The dominant paradigm for the evolution of mutator alleles in bacterial populations is that they spread by indirect selection for linked beneficial mutations when bacteria are poorly adapted. In this paper, we challenge the ubiquity of this paradigm by demonstrating that a clinically important stressor, hydrogen peroxide, generates direct selection for an elevated mutation rate in the pathogenic bacterium *Pseudomonas aeruginosa* as a consequence of a trade-off between the fidelity of DNA repair and hydrogen peroxide resistance. We demonstrate that the biochemical mechanism underlying this trade-off in the case of *mutS* is the elevated secretion of catalase by the mutator strain. Our results provide, to our knowledge, the first experimental evidence that direct selection can favour mutator alleles in bacterial populations, and pave the way for future studies to understand how mutation and DNA repair are linked to stress responses and how this affects the evolution of bacterial mutation rates.

## 1. Introduction

A minority of bacteria isolated from natural and clinical environments have genetically elevated mutation rates, often as a result of mutations in genes involved in the highly conserved methyl-directed mismatch repair (MMR) pathway [1]. Computer simulations and experiments have shown that mutator alleles can spread in bacterial populations as a result of second-order selection [2–6]; that is, selection drives an increase in the frequency of beneficial mutations, and mutator alleles increase in frequency when they are linked to these beneficial mutations. According to this paradigm, stress, which we define as any environmental perturbation that decreases bacterial growth rate, indirectly drives an increase in mutator frequency by generating selection for stress-resistance mutations. While stress affects indirect selection for mutators, stress can also cause phenotypic changes, which, in turn, are likely to alter selection for mutation rates. Of particular note is the fact that stress itself can result in phenotypic increases in mutation rate [7–9]. For example, many stressors, including antibiotics, induce the expression of stress response pathways that are associated with an increase in the mutation rate, by inducing the expression of alternative, low-fidelity DNA polymerases [10]. This may constrain or promote the evolution of genetic mutators, depending on how the stress differentially affects phenotypic mutation rates of mutators and wild-type. The frequency of mutator alleles in natural and clinical populations of pathogenic bacteria provides a good example of this expected association between stress and mutator prevalence. Mutator alleles are rare in bacteria isolated from environmental samples and acute infections [11,12], but mutator prevalence is very high in populations isolated from chronic infections that are subjected to recurrent strong stresses, in the form of antibiotic treatments [11,13].

Independent of any linkage to deleterious or beneficial mutations, the simplest form of selection that acts on mutator alleles is direct selection that stems from the physiological costs and benefits of mutators alleles. For example, recent study suggests that defective DNA oxidative repair (GO), or MMR systems in bacteria make cells more sensitive to oxidative stress, ultraviolet light or temperature [14,15]. Moreover, it has been demonstrated that an *Escherichia coli mutS* mutant displays altered expression of a small number of housekeeping genes [16], raising the possibility that direct fitness costs and benefits may be associated with mutator alleles as a result of the pleiotropic effects of mutator alleles on gene expression.

While the original goal of this study was to investigate the interplay between phenotypic and genetic changes in mutation rates in response to stress, preliminary findings led us to study the impact of direct stress-imposed selection on mutator evolution. We used a model system involving oxidative stress in wild-type and mutator strains of *Pseudomonas aeruginosa*. Reactive oxygen species (ROS) damage DNA, membranes and proteins, and are thought to be a highly relevant stress for pathogenic bacteria because both antibiotics and the immune system generate ROS as a mechanism for killing bacterial pathogens [17,18]. In this study, we test for direct selection on a number of mutator alleles in the presence of stress by measuring the survivorship after exposure to a lethal dose of hydrogen peroxide. In contrast to previous study, we find resistance to oxidative stress correlates positively to mutation rate in the absence of stress, suggesting the existence of direct benefit of mutator alleles. At a mechanistic level, we find that this increase in resistance is attributable to elevated levels of catalase secretion, at least by the *mutS* mutator. Exposure to oxidative stress causes an increase in mutation rate in the wild-type strain, but mutators do not display stress-induced increases in mutation rate as a result of their intrinsic resistance to hydrogen peroxide. Direct competition experiments between mutators and wild-type confirm the nature of ‘public good’ of the protective enzymes, as both strains share the benefits when exposed to the stress.

## 2. Results

### (a) Mutation rate and stress resistance

**Figure 1.**
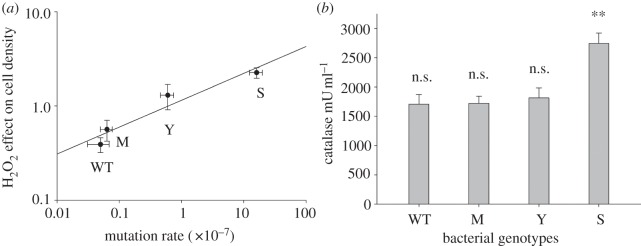
Mutation rate, hydrogen peroxide resistance and catalase activity of mutators and wild-type (WT). (*a*) Shows the correlation between mutation rate, as measured in the absence of exposure to hydrogen peroxide, and hydrogen peroxide resistance, measured as relative change in cell density following exposure to hydrogen peroxide relative to untreated controls (values >1 mean an increase in cell density after treatment with hydrogen peroxide, and values <1 represent a negative effect of hydrogen peroxide on cell growth, relative to cells treated with H_2_O). (*b*) Bars show the catalase activity (mU of enzyme per ml) in the supernatant of the different *Pseudomonas* cultures prior to exposure to hydrogen peroxide as measured using the Amplex red reagent-based assay. Significant differences are represented by asterisks (*p ≤ 0.05, **p ≤ 0.01 and ***p ≤ 0.001, respectively) when suitable and not significant (n.s.) when not. Plotted data show the mean of (*a*) eight or (*b*) five independent replicates, error bars represent s.e.m.

To test for direct selection on mutator alleles, we measured the mutation rate and hydrogen peroxide resistance of a wild-type strain of *P. aeruginosa* and isogenic mutator strains carrying deletions in DNA repair pathways that are commonly isolated in natural populations (*mutS*, *mutY*, *mutM*). Consistent with previous study, we find that these mutations lead to small (*mutM*), moderate (*mutY*) and large (*mutS*) increases in the mutation rate. Note that in these assays, mutation rate was measured prior to exposure to hydrogen peroxide. Surprisingly, we find that mutator strains are intrinsically more resistant to hydrogen peroxide than the wild-type strain and that hydrogen peroxide resistance shows a strong positive correlation to the mutation rate (figure 1*a*; linear correlation, *r* = 0.9777, d.f. = 3, *p* = 0.0222). Because we measured hydrogen peroxide resistance over a short time period (15 min of exposure) and the strains were constructed *de novo* for this study, we can be confident that the increased resistance of mutators reflects intrinsic resistance of mutator strains, and not an increase in the rate of evolution of resistance in mutator strain. These results demonstrate the existence of a direct benefit associated with mutator alleles in the presence of oxidative stress as a result of a trade-off between DNA repair efficiency and hydrogen peroxide resistance.

One of the most important mechanisms of hydrogen peroxide resistance is the secretion of extracellular enzymes, such as catalase, that degrade hydrogen peroxide before it has the chance to damage the cell. To investigate the causes of the oxidative stress resistance in the mutator strains, we analysed the catalase activity in the supernatant of our wild-type and mutator strains. Mutator strains showed levels of catalase secretion non-significantly different from the wild-type (figure 1*b*; Kruskal–Wallis *χ*_1_ = 1.69, *p* = 0.1936), except for *mutS*, which produced the highest amount of catalase, around 60 per cent more than the wild-type (Kruskal–Wallis *χ*_1_ = 6, *p* = 0.0143). This suggests that an elevated catalase secretion is the biochemical mechanism underlying the hydrogen peroxide resistance in the case of *mutS*. It is possible that the *mutY* and *mutM* strains have elevated hydrogen peroxide mechanisms as a result of the upregulation of alternative pathways for hydrogen peroxide resistance found in *P. aeruginosa* [19]; alternatively, it is possible that these mutators have elevated catalase secretion, but that our assay lacked the power to detect the difference in catalase secretion between these weak mutators and the wild-type.

### (b) Stress and mutation rate

**Figure 2.**
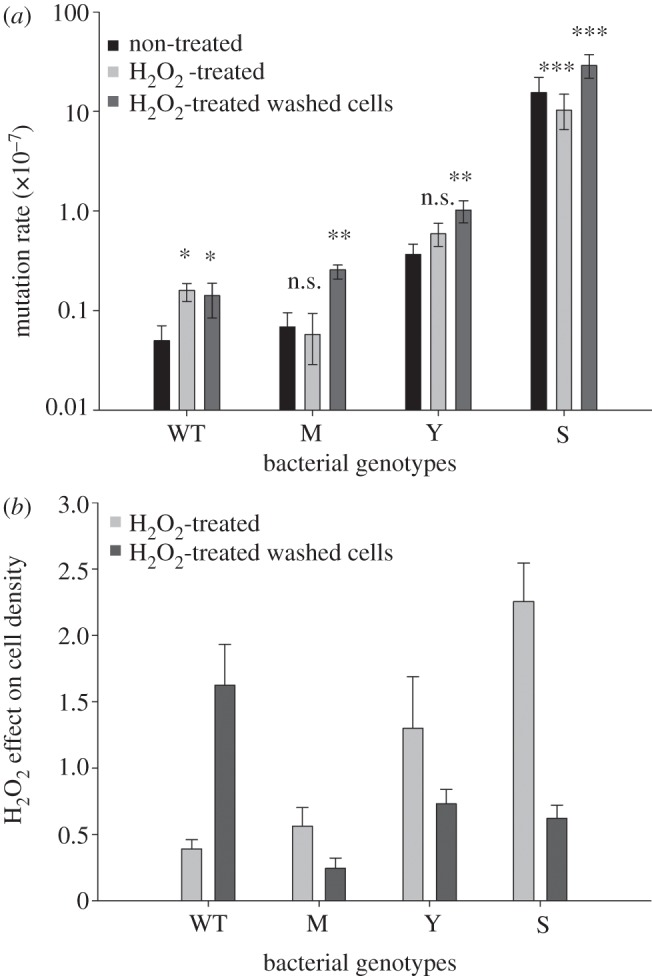
Mutation rate and hydrogen peroxide effect on cell density in mutators and wild-type (WT). (*a*) Shows mutation rate levels of *P. aeruginosa* strains (black bars) and effect of hydrogen peroxide treatment on mutation rate in non washed and washed cells (light and dark grey bars). Significant differences between untreated and both treatment are represented (n.s., *p > 0.05, **p ≤ 0.05, ***p ≤ 0.01 and 0.001, respectively). Error bars are confidence intervals as calculated using the Ma-Sandri-Sarkar maximum-likelihood method. In (*b*), hydrogen peroxide effect on cell density in mutators and WT not washed or washed, compared with cells treated with H_2_O (>1 positive effect on growth; <1 negative effect). Error bars are s.e.m. For both panels, eight replicates were used.

To measure the impact of stress on the mutation rate of wild-type and mutators, we measured the mutation rate after exposure to hydrogen peroxide (figure 2*a*). Consistent with previous studies, we found that the wild-type strain showed a significant increase in mutation rate following exposure to hydrogen peroxide (*t*_14_ = 2.43, *p* < 0.05). Mutators, on the other hand, showed a one-third-fold decrease in mutation rate in the case of the strong mutator (*mutS*; *t*_14_ = 4.19, *p* < 0.001) and no significant change for the mild and weak mutators. The consequence of this is that the increase in mutation rate associated with strong mutator alleles is dependent on stress.

The simplest explanation for why hydrogen peroxide does not increase the mutation rate in mutator strains is that mutators are intrinsically hydrogen peroxide resistant. To directly test this hypothesis, we measured the impact of exposure to hydrogen peroxide on cell growth and mutation rate on cell suspensions that had been washed and resuspended in saline buffer prior to being exposed to hydrogen peroxide, effectively eliminating protection against hydrogen peroxide by secreted catalase (figure 2*a*,*b*). Consistent with previous studies [8,20], we find that cell suspensions of wild-type cells are more resistant to oxidative stress than mutators: cell density after the hydrogen peroxide treatment decreased around 40 per cent, 30 per cent and 75 per cent for *mutS*, *mutY* and *mutM*, respectively, whereas wild-type was 100 per cent resistant (figure 2*b*). By contrast, hydrogen peroxide treatment was benign for *mutS* and *mutY* but decreased cell density in *mutM* and wild-type around 40 per cent and 60 per cent, respectively (figure 2*b*). The decrease in the hydrogen peroxide tolerance of mutator cell suspensions was accompanied by a significant increase in the mutation rate (figure 2*a*), suggesting that the mutation rate of mutators does not increase in the presence of supernatant because mutator populations are resistant to hydrogen peroxide. This provides further support that the mechanism underlying the increased hydrogen peroxide resistance of mutators is that they secrete protective enzymes.

Given that mutator resistance to hydrogen peroxide appears to be the result of secreted exoproducts, we hypothesized that the wild-type strain have a comparable fitness to the *mutS* mutator when cultured together, as opposed to lower fitness as monocultures described earlier. Consistent with this hypothesis, we found no fitness differences between the mutator and the wild-type when cultured together (see the electronic supplementary material, figure S1, ANOVA *F*_2_ = 2.57, *p* = 0.0931), or between the hydrogen peroxide treated and the untreated cultures (ANOVA, *F*_1_ = 0.13, *p* = 0.7227), with mean fitness minimally higher in treated cultures (1.0152 ± 0.0336 compared with 1.0014 ± 0.0346). These results reinforce the idea that mutators are not more sensitive to oxidative stress than the wild-type, and that they can have a direct fitness advantage at least in the short term.

## 3. Conclusions

Both theoretical and empirical study demonstrates that second-order selection plays a crucial role in the evolution of bacterial mutation rates [2–4]. Here, we show that mutators can also be favoured by direct selection as a result of oxidative stress resistance; a major advantage for an opportunistic pathogen such as *P. aeruginosa*.

Surveys of populations of bacterial pathogens have revealed a clear link between environmental stress mediated by the immune system and antibiotics, and the prevalence of mutators. For example, a substantial fraction of strains of *P. aeruginosa* isolated from long-term chronic infections have an elevated mutation rate while mutators are undetectable in samples isolated from short-term acute infections [21], and similar trends have been documented in other species [1]. The most surprising feature of our results is that we find mutations that elevate the mutation rate by compromising DNA repair are associated with a direct fitness benefit, in terms of increased resistance to oxidative stress. Our results strongly suggest that the increased resistance of mutators to hydrogen peroxide is directly attributable to an increased secretion of enzymes that protect against hydrogen peroxide. *Pseudomonas aeruginosa* antioxidant defences are extraordinary and relatively complex compared with other bacteria, including superoxide dismutases, alkyl hydroperoxide reductases and three catalases [22,23]. Among the catalases, *P. aeruginosa* KatA has unique properties such as high stability, high specific activity and extracellular presence [19]. Here, we propose that increased production of extracellular KatA is responsible for the high resistance to hydrogen peroxide observed, at least for the *mutS* strain, a result that does not contradict previous study showing that intracellular catalase activity does not differ between wild-type and *mutS* strains of *P. aeruginosa* [20]. Although we do not find evidence of higher catalase activity for *mutY* and *mutM*, our results indicate the enhanced production of some extracellular protective enzyme for these strains too. In agreement with previous study [8,20], we find that removing extracellular products by measuring resuspended cell pellets is associated with an increased sensitivity of mutators cells to hydrogen peroxide and an increase in hydrogen-peroxide-induced mutagenesis. We suggest, however, that the more biologically and ecologically relevant method for assaying bacteria stress resistance is to measure cell phenotypes, such as stress resistance, in the presence of secreted proteins, as bacteria secrete a large number of proteins that can mediate a range of ecologically important effects [24]. Surprisingly, wild-type strains are stress resistant when the supernatant is removed (but susceptible when present). We speculate that this could be connected to the higher iron availability in the culture media compared with the washed cells that enhances lethal hydroxyl radical formation inside the cells [25].

The mechanistic link between DNA repair-deficiencies and elevated extracellular enzyme production is unclear. Micro-organisms display complex and highly integrated responses to a wide range of stressors that often involve changes in the expression in a substantial portion of genome, resulting in profound alterations in cellular physiology and behaviour [9]. Exposure to a wide range of stressors has been demonstrated to result in an increase in the mutation rate owing to the induction of expression of alternative, low-fidelity DNA polymerases or the repression of DNA repair enzymes [7,10]. It is possible that the level of DNA damage sensed by the DNA-repair-deficient strains could increase production of protective enzymes, for example through the SOS response (a bacterial stress response pathway that is expressed in response to DNA damage), although there is no current evidence of significantly higher level of double-strand breaks on a *mutS* strain compared with the wild-type, at least for *E. coli* [16]. Another study has reported results that are consistent with this view: Maisnier-Patin *et al.* [26] found that lines of *Salmonella typhimurium* that were allowed to randomly accumulate mildly deleterious mutations consistently upregulated the expression of chaperones, providing good evidence that increased chaperone expression occurred as a consequence of a high mutational load. Intriguingly, this study demonstrated that elevated chaperone expression helped to buffer against the deleterious effects of random mutations, demonstrating that responses to mutation induced stress can have important evolutionary consequences. Owing to the time frame of our experiments, it seems unlikely that the upregulation of protective enzymes is directly associated with the mutational load, but we suggest that there is a link with the phenotype of the functionally different deleted genes.

Studies of infecting populations of *P. aeruginosa* are consistent with the idea that oxidative stress generates direct selection for compromised DNA repair. ROS released by polymorphonuclear lymphocytes constitute an important first line of defence against bacterial pathogens and mutator prevalence in populations of *P. aeruginosa* from cystic fibrosis patients who suffer from chronic infections correlates positively to the presence of markers of oxidative stress [13]. However, previous study suggesting that mutator bacteria are more sensitive than the wild-type variants to different stresses make it difficult to explain why mutators are not quickly disfavoured by selection [27]. By contrast, a number of recent high-throughput screens for genes involved in intrinsic antibiotic resistance have shown that mutator mutants tend to be associated with increased resistance to a wide range of antibiotics [28], many of which generate oxidative stress [25]. This raises the intriguing possibility that the strong association that is observed between antibiotic use and mutator prevalence in bacterial populations [21,29] may also be partially attributable to the direct benefits associated with mutator alleles.

Although theoretical models of mutator evolution have not considered the possibility that mutator alleles are associated with a direct benefit, simple arguments suggest that direct selection may play a key role in increasing the efficacy of second-order selection on mutator alleles. Mutator invasion success is typically positive frequency dependent [30,31], as the number of beneficial mutations generated by a mutator lineage relative to a wild-type lineage will increase with the frequency of the mutator lineage. Importantly, a number of studies have demonstrated that mutators can invade from rare frequencies if selection is strong [32,33]. A direct benefit for mutator alleles stemming from increased stress resistance may play a pivotal role in the evolutionary dynamics of mutator alleles by helping mutators to reach sufficient frequencies to be favoured by second-order selection. Interestingly, if mutators are producing catalase as a ‘public good’, then this may also contribute to explaining advantage of mutators in structured populations, where benefits of public good are more likely to be received by other mutators [24]. The ‘public good’ characteristics of the secreted protective enzymes could also explain the persistence of wild-type strains as illustrated by the competition experiments carried out in this study, where the wild-type strain does not show a lower fitness than mutators in the presence of hydrogen peroxide.

A trade-off between DNA repair and stress resistance may also help to maintain elevated mutation rates over the long-term by coupling decreased mutation rates to reduced stress resistance. However, fitness trade-offs can be overcome to some extent by selection given previous study showing the apparent elimination of trade-offs associated with ecological specialization [34] and antibiotic resistance [35]. In addition, there is evidence that indirect selection against mutator strains owing to accumulation of rare deleterious mutations is strong [27,36].

## 4. Material and methods

### (a) Bacterial strains and media

The strains used in this study are wild-type *P. aeruginosa* PAO1 (WT): the mismatch repair-deficient strain *ΔmutS* (*mutS*) and the DNA oxidative repair-system-deficient strains *ΔmutY* (*mutY*) and *ΔmutM* (*mutM*). The last three strains were constructed from the isogenic *P. aeruginosa* PAO1 de novo, especially for this study, using the *Cre-lox* system for gene deletion and antibiotic resistance marker recycling following previously described protocols [37,38]. The three mutator strains have a *aac1* cassette conferring resistance to gentamicin (15 μg ml^−1^).

All bacteria were cultured on 5 ml Kings–Broth (KB) medium microcosms at 37°C shaking at 250 rpm. M9 medium was used to dilute the cultures when necessary.

### (b) Hydrogen-peroxide-induced mutation rate

Mutation rate was calculated using a fluctuation test as described previously [39]. Eight independent cultures per treatment and strain (WT, *mutS*, *mutY* and *mutM* and evolved *mutS* populations) were set up diluting 10^−6^ fold precultures from single colonies. To investigate the effect of hydrogen peroxide on mutation rate, 200 μl of each culture was treated with hydrogen peroxide (Sigma-Aldrich) at a concentration of 75 mM for 15 min at 37°C in 96-well plates. Control cultures were treated with H_2_O under identical conditions.

After the treatment, direct volumes of the eight replicates were spread into KB agar plates with the antibiotic rifampicin (100 μg ml^−1^) and incubated at 37°C for 24 h. Appropriate dilutions were plated onto KB agar plates to count the total number of cells of the same cultures after incubation overnight. Estimation of mutation rate values and confidence intervals was carried out using Ma-Sandri-Sarkar maximum-likelihood estimator method as implemented in www.mitochondria.org/protocols/FALCOR.html [40].

To allow direct comparison with previous study, we measured parental strains mutation rate and the relative change in cell density after treatment with hydrogen peroxide (75 mM, 15 min at 37°C) washing the cells in 0.8 per cent NaCl as described in Sanders *et al*. [20].

### (c) Hydrogen peroxide effect on cell density

To calculate hydrogen peroxide effect in the growth of the different strains, after treatment of eight replicate cultures as explained before, we measured viable cells using BacTiter-Glo Reagent (Promega) as detailed by the manufacturer. Change in cell density of each strain was calculated as viable cells after treatment of 15 min with 75 mM hydrogen peroxide relative to viable cells in H_2_O-treated controls. The response variable in figures is the ratio between treatments (cell density treated with hydrogen peroxide divided by cell density treated with H_2_O) and are in the form of a proportion. Values greater than 1 mean an increase in cell density after treatment with hydrogen peroxide, and values less than 1 represent a negative effect of hydrogen peroxide in cell growth, relative to cells treated with H_2_O.

### (d) Catalase activity assay

We used the Amplex red catalase assay kit from Molecular Probes, as recommended by the manufacturer. Five individual samples of the supernatant of every strain (WT, *mutS*, *mutY* and *mutM* and *mutS*) were analysed. Bacterial cultures were normalized by optical density_600_ (OD_600_). Measurements were made in the SpectraMax M2 microplate reader (Molecular Devices).

### (e) Competitive fitness assay

Competition experiments between *P. aeruginosa* PAO1 wild-type and mutator (GmR) strains were carried out in KB microcosms. Equal volumes of 0.5 OD cultures of each mutator strain were combined with the WT, mixed and appropriate dilutions plated in KB with and without gentamicin (15 μg ml^−1^). Six independent cultures per three combinations were treated with hydrogen peroxide or H_2_O and transferred immediately into a microcosm with fresh media in a 10^−2^ dilution. After overnight growth, cultures were diluted and plated onto KB plates with and without gentamicin; only mutators can grow on the latter. Fitness of mutators was calculated as the ratio of mutator to wild-type malthusian parameters [41].
